# Maintaining routine HIV and tuberculosis testing services in sub-Saharan African countries in the context of COVID-19: Lessons learnt and opportunities for improvement

**DOI:** 10.4102/ajlm.v10i1.1413

**Published:** 2021-06-17

**Authors:** Collins O. Odhiambo, Anafi Mataka, Marguerite Massinga Loembe, Pascale Ondoa

**Affiliations:** 1 African Society for Laboratory Medicine, Addis Ababa, Ethiopia; 2 Laboratory Division, Africa Centres for Disease Control and Prevention, Addis Ababa, Ethiopia; 3 Amsterdam Institute for Global Health and Development, Department of Global Health, University of Amsterdam, Amsterdam, the Netherlands

Since being declared a public health emergency of international concern on 30 January 2020, the coronavirus disease 2019 (COVID-19) has spread internationally, reaching the stage of a global pandemic.^1^ African countries quickly put in place social and public health measures to limit the spread of the disease, with some of the most ‘visible’ measures being lockdowns, physical distancing and the overall surge of healthcare services to support the COVID-19 response.

The Director-General of the World Health Organization, calling for increased testing, recommended ‘test, test, test’ as a critical step to contain the spread of the disease.^2^ When the first African case was reported in Egypt in February 2020, only two centres of excellence laboratories on the continent were capable of conducting severe acute respiratory syndrome coronavirus 2 polymerase chain reaction testing, the gold standard assay recommended by the World Health Organization. One key strategy to swiftly scale-up the testing capacity for severe acute respiratory syndrome coronavirus 2 has been the re-tooling of existing nucleic acid amplification testing platforms toward the control of COVID-19. Nucleic acid amplification testing platforms available from HIV and tuberculosis control programmes offered a unique opportunity given their large footprint across several countries and the availability of in-service training programmes for the operators. At least three COVID-19 molecular diagnostic assays listed under the emergency use authorisations of the United States Food and Drug Administration or the World Health Organization can be used on platforms commonly available within the HIV and tuberculosis programmes – the Abbott m2000 RealTime System, Cepheid’s GeneXpert^®^ system, and the Roche Cobas 6800/8800 system.^3^

Although COVID-19 testing volumes in most African countries remain below or at the lower threshold required for relaxing the containment measures (10–30 tests per confirmed case),^4^ have we gone a step too far in refocusing most of the laboratory capacity to COVID-19 testing? Mounting evidence suggests that the ‘covidisation’ of the healthcare system and, more specifically, diagnostics poses a risk to maintaining other routine testing services for the control and prevention of endemic diseases like HIV, malaria, syphilis and tuberculosis.^5^ Additionally, lockdowns imposed in most countries prevent patients from accessing healthcare and this may result in increased treatment failure among those on medication, and increased maternal and child mortality in the context of poorly attended childbirth and reduced antenatal care. Without intervention, there is a risk of reversing many of the gains achieved towards meeting the targets of the Joint United Nations Programme on HIV/AIDS 95:95:95, the End TB Strategy, and the United Nations Sustainable Development Goals, including ending the ‘big three’ diseases – HIV, tuberculosis and malaria. HIV, tuberculosis and malaria programmes have made substantial progress towards achieving global targets due to massive donor funding. However, the Joint United Nations Programme on HIV/AIDS estimates that there could be hundreds of thousands of extra deaths from HIV if routine services, including HIV screening, viral load and early infant diagnosis, are disrupted.^6^

Eighty-five percent of national-level respondents from 61 countries participating in a World Health Organization, United Nations Children’s Fund and Global AIDS Vaccine Initiative poll reported lower vaccination proportions in May 2020 compared to the level in January 2020 – February 2020.^7^ Additionally, disruption of maternal and child health services is estimated to contribute to at least 8% more deaths per month.^8^ A survey of the Global Fund’s supported programmes across 106 countries revealed that at least 85%, 80% and 76% of HIV, tuberculosis and malaria control services, respectively have been disrupted by the COVID-19 pandemic.^9^ A recent modelling analysis from the STOP TB partnership further illustrated how an additional lockdown period of three months could lead to 6.3 million more cases of tuberculosis globally by 2025, causing a setback of at least five years on the progress achieved thus far.^10^ More specifically, the survey revealed that at least 20% of HIV and tuberculosis laboratory services are facing high disruption with much of the advanced diagnostic equipment repurposed and the workforce reassigned for COVID-19 testing. A World Health Organization survey of essential services revealed that HIV testing (*n* = 38) and viral load monitoring (*n* = 23) were the two most frequently interrupted testing services in the 61 countries that responded.^11^ It is important to note that laboratory testing is key to identifying disease cases, breaking transmission chains and monitoring patients on treatment.

In outbreak situations, healthcare stakeholders at the national level must be able to quickly address testing needs and, at the same time, identify the testing threshold needed to ensure that routine testing services are maintained and health targets for the control of essential diseases remain on track. However, data about the overall function of health services are usually fragmented and rarely realtime on the continent. Furthermore, movement restrictions have further hampered the collection of information on the continuation of essential healthcare services, particularly diagnostics.

The African Society for Laboratory Medicine through the Laboratory Systems Strengthening Community of Practice (LabCoP)^12^ conducted an online survey (June 2020) among LabCoP member countries to seek a deeper understanding of the disruptions in HIV and tuberculosis testing services during COVID-19 diagnosis scale-up. The LabCoP is funded by the Bill and Melinda Gates Foundation and is composed of multidisciplinary teams (clinicians, laboratorians and civil society) with strong collaboration with the ministries of health from 14 sub-Saharan African countries.^12^ Consensus responses to the survey questionnaire were received from the multidisciplinary teams of 10 of the 14 countries. Among those, nine countries reported a decline in viral load testing volumes, eight reported a decline in tuberculosis GeneXpert testing volumes, while seven reported a decline in early infant diagnosis volumes during the COVID-19 response, thereby confirming the Global Fund survey findings.^9^ In Uganda, one of the few LabCoP participant countries with a publicly available viral load dashboard for testing services, there was a 28% reduction in viral load test volume (from 104 474 to 74 841 tests) between March 2020, when the first COVID-19 case was reported in Uganda, and May 2020.^13^ The main reason provided by most countries (9 of 10 countries) for the decline in testing volumes was either the inability of patients to visit the health facilities to access HIV and tuberculosis services due to movement restrictions aimed at limiting COVID-19 transmission (at least 42 African countries had imposed partial or complete lockdowns as of May 2020) or patient fear of contracting the disease while visiting health facilities. Five countries reported stock-outs of testing kits, reagents and laboratory consumables as a result of border closures. Four countries indicated that the surge in COVID-19 testing volumes caused or exacerbated the shortage of healthcare workers, as they were reassigned to support the COVID-19 response (especially personnel skilled in molecular testing), thereby affecting the provision of other essential diagnostics based on polymerase chain reaction technology. Whereas 8 of 10 countries reported re-purposing between 25% to 83% of the total HIV and tuberculosis testing equipment capacity in-country for COVID-19 testing, only two countries identified insufficient molecular testing capacity as a barrier to maintaining HIV and tuberculosis diagnostic services. Two separate analyses indicated that most HIV and tuberculosis instruments are often operated below their full capacity,^14,15^ indicating that available instruments in most countries may be sufficient to support both COVID-19 and HIV and/or tuberculosis testing. Moreover, under the impulse of strong HIV and tuberculosis disease control programmes funded by the United States President’s Emergency Plan For AIDS Relief and the Global Fund, the equipment is used almost exclusively for one disease area due to vertical programming, despite the instruments’ multiplexing capability and the recommendation to ‘integrate’ testing.^16^ Additionally, many of the countries who either repurposed HIV and/or tuberculosis equipment for COVID-19 testing or refocused testing still experience challenges in long turn-around times for results, quality assurance and procurement issues, among others, indicating systemic weaknesses that need attention.

The difficulty in scaling the COVID-19 response while maintaining routine testing services for other essential diseases indicates that many countries are still struggling to achieve a multipurpose, resilient and effective laboratory network to address the needs of clinical and surveillance testing services and meet the prevention and control targets for all essential diseases.^17^ With more than a decade of large initiatives aimed at laboratory system capacity building in Africa, what are we still missing to ensure that laboratory networks can more effectively forecast and organise laboratory services in both routine and emergency situations?

Through the LabCoP country annual self-assessment reports, we have been able to identify some of the inherent weaknesses in the viral load testing cascade, including laboratory network optimisation and monitoring and evaluation (M&E). Laboratory stakeholders at the central level often do not have a comprehensive overview of available resources and capacities across the network, or how these can be leveraged to strengthen the various functions of the laboratory system; for example, how higher-tier laboratories can organise external quality assessment for those in lower tiers. Further, at the laboratory level, managers and directors often lack the managerial skills and leadership needed to organise and implement comprehensive laboratory management systems that enable laboratories to conduct the tiered functions of the network for multiple diseases; these include knowledge in determining staffing levels, testing capacity, and roles and responsibilities, as well as skills in the use of geographic information system tools like LabMap^18^ and LabReady to map, analyse and optimise the laboratory network capacity, functions and services. An inclusive approach that fosters strong collaboration across sectors (i.e., public and private, academic, civilian and military, human and animal health, vertical disease programmes, etc.), as well as an evidence-based decision-making process based on available laboratory data, should be used to inform scale-up of testing for COVID-19 and any emerging disease. Our survey has highlighted that evidence was seldom leveraged to inform scale-up of COVID-19 diagnostic services; only one of three countries that reported the procurement of additional molecular testing equipment to support the surge in COVID-19 testing identified a shortage of testing platforms as the reason for the reduction in HIV/tuberculosis testing, whereas only two of the four countries that reported hiring additional laboratory staff identified insufficient staff capacity as a gap.

To address some of these gaps, the African Society for Laboratory Medicine is working with various stakeholders, including the Association of Public Health Laboratories, the Clinton Health Access Initiative, the Foundation for Innovative New Diagnostics, the World Health Organization, and the United States Centers for Disease Control and Prevention, among others, to develop a leadership and mentorship programme focused on leading and managing tiered laboratory networks to offer clinical care and public health services during routine and emergency situations. A guidance document for scaling up COVID-19 diagnosis within the laboratory network while maintaining essential testing has been developed.^19^ Additionally, the African Society for Laboratory Medicine is convening an M&E sub-community of practice of the LabCoP articulated around the delivery of a fit-for-purpose M&E training and mentoring curriculum with the opportunity for direct technical assistance to LabCoP country teams. The key outcome of this initiative is the development or improvement of national dashboards to monitor and evaluate the implementation and performance of the HIV viral load testing cascade and diagnosis of other diseases, including COVID-19. Additionally, it is anticipated that the multi-country discussion will highlight best practices and identify actual needs that will inform the update of the current guidance document for establishing an M&E framework.^20^

The difficulty of scaling up COVID-19 diagnostics while maintaining routine testing of HIV, tuberculosis and other essential diseases relates to pre-existing laboratory systemic weaknesses. Solutions to durably tackle the gaps crippling diagnostic services include better knowledge, management and optimisation of national tiered networks to become more resilient in the face of health emergencies. Attaining skills in the management of laboratory networks and strengthening of M&E systems in-country will contribute to better and faster identification of strengths and bottlenecks and mutualisation of existing resources across the laboratory network for routine and emergency testing needs. Additionally, robust political commitment and sufficient provision of domestic funding are needed to ensure that all local health needs are adequately covered and are not compromised when addressing global priorities.
